# Differences in the growth of microorganisms depends on the type of semi-solid enteral nutritional supplements

**DOI:** 10.1186/s40780-023-00297-8

**Published:** 2023-09-01

**Authors:** Sachiko Omotani, Kanaha Murakami, Arisa Naka, Yasutoshi Hatsuda, Michiaki Myotoku

**Affiliations:** Faculty of Pharmacy, Osaka Ohtani University, 3-11-1, Nishikiori-Kita, Tondabayashi, Osaka, 584-8540 Japan

**Keywords:** Semi-solid enteral nutrition, RTH preparations, Microorganisms

## Abstract

**Background:**

Enteral nutritional supplements are used in many medical facilities and home care, but require appropriate management because they are nutrient-rich products. Recently, infection control methods for Ready To Hang (RTH) preparations, which are widely used and are expected to reduce the risk of infection, have not been established in Japan and are dependent on caregivers. Therefore, we evaluated the difference in the growth of microorganisms depending on the type of enteral nutrients following contamination with microorganisms.

**Methods:**

Nine types of enteral nutrition were used. *Escherichia coli* (*E. coli*) W3110, *Serratia marcescens* (*S. marcescens*) NBRC3046, and *Candida albicans* (*C. albicans*) IFM61197 were used as test bacteria. The bacterial solution was added to the enteral nutritional supplement, adjusted, and the number of bacteria was measured at 0, 4, 8, and 24 h after the addition of the bacterial solution at 25 °C and in the dark.

**Results:**

*E. coli* and *S. marcescens* grew in RACOL^®^-NF SemiSolid for Enteral Use, Hine_®_ Jerry AQUA, and Mermed Plus_®_ over a 24-h period; however, a decrease was observed for other enteral nutrition products. In contrast, *C. albicans* grew in all enteral nutrition products.

**Conclusion:**

Because the viscosity and calorie content vary among enteral nutrition preparations in which growth was observed, we found that pH had the greatest effect on the differences in bacterial growth. Nonetheless, *C. albicans* growth occurred in all nine types of enteral nutrients, indicating that unlike bacteria, its growth was independent of pH. If semi-solid enteral nutrients are contaminated with microorganisms for any reason, microorganisms will grow, so appropriate infection control is necessary to prevent infection.

## Background

The number of elderly people aged 65 and over in Japan has reached a record high at approximately 29.1% of the total population (in 2022) [1]. In 2021, Japan will have the highest percentage of people aged 65 and over, making it a super-aged society; therefore, countermeasures against malnutrition among the elderly have become an issue. Enteral nutrition (EN) is recommended for the nutritional management of patients who have difficulty in oral intake and who have preserved intestinal function [2, 3]. In addition, home medical care in Japan is being promoted and the number of patients undergoing enteral feeding at home has increased.

EN may be divided into oral nutrition and tube feeding depending on the route of administration. For tube feeding, nutrients are injected into the nose or stomach through a catheter. Gastroesophageal reflux disease, aspiration pneumonia, and diarrhea are complications of conventionally used liquid EN. In Japan, the use of semi-solid EN has gained popularity [4, 5] in recent years, because prevents the complications observed with liquid EN [4–8]. It also reduces the burden on caregivers, because it can be administered in a shorter time [9–11]; however, semi-solid EN, which is more viscous than liquid nutrition, readily adheres to the lumen of the catheter and is considered a cause of catheter contamination and infection. The Guidelines for Parenteral and Enteral Nutrition, 3^rd^ Edition, indicate that the administration of enteral nutritional products that are dissolved or diluted should be completed within 8 h to minimize microbial growth and within 24 h for Ready To Hang (RTH) preparations [3]. RTH preparations are frequently used in medical facilities and home care because they are associated with reduced contamination, the product is not transferred into a container and there is little risk of contamination from the bag itself [3, 12]. Nutritional management of home patients is often done by family members as well as medical personnel; however, the methods are not standardized and there are few reports regarding the importance of properly handling enteral nutritional supplements. In addition, a variety of semi-solid EN products are on the market and it is likely that differences in ingredients and physical properties affect infection control.

Our study aimed to investigate how the characteristics of EN products affect the growth of microorganisms. We determined the difference in the growth of microorganisms based on the type of EN when semi-solid EN of RTH preparations was contaminated with microorganisms. Our study aimed to investigate how the characteristics of EN products affect the growth of microorganisms.

## Materials and methods

### Microorganisms employed

The bacterial strains used in the study were *Escherichia coli* (*E. coli*) W3110, *Serratia marcescens* (*S. marcescens*) NBRC3046, and *Candida albicans* (*C. albicans*) IFM61197. *C. albicans* IFM 61197 was obtained from the National BioResource Project (http://www.nbrp.jp/). *E. coli*, *S. marcescens* and *C. albicans* could be a problem in healthcare-associated infections. *E. coli* and *S. marcescens* have been reported to bacteria becoming drug-resistant in nursing homes that use gastrostomy tubes [13]. *C. albicans* has also been reported to form biofilms that reduce the efficacy of drugs and make treatment difficult [14]. *Staphylococcus aureus* (*S. aureus*) is a very important bacterium in infection diseases. However, we used *E. coli*, *S. marcescens* and *C. albicans* in the experiment since growth of *S. aureus* and coliforms in EN had already been researched in other reports [15, 16].

### Test solutions

Nine semi-solid enteral nutritional products were used: (A) RACOL^®^-NF SemiSolid for Enteral Use (EN Otsuka Pharmaceutical Co., Ltd. JAPAN), (B) Hine_®_ Jerry AQUA (Otsuka Pharmaceutical Factory, Inc. JAPAN), (C) Mermed Plus_®_ (Terumo Corporation, JAPAN), (D) Medif_®_ Push Care_®_ 2.5 (Nestlé Health Science, JAPAN), (E) ISOCAL^®^ SemiSolid Support (Nestlé Health Science, JAPAN), (F) PG soft EJ_TM_ (Terumo Corporation, JAPAN), (G) PG soft A_TM_ (Terumo Corporation, JAPAN), (H) PG Soft MP_TM_ (Terumo Corporation), and (I) F2 Shot EJ_TM_ (Terumo Corporation, JAPAN). Table 1 shows the ingredients contained in each product.

**Table 1:** The composition of enteral nutrition products

No	Product Name	Company	Concentration (kcal/g)	pH	Osmotic Pressure (mOsm/L)	Viscosity (mPa・s)	Carbohydrate (mg/g)	Glucose (mg/g)	Dietary Fiber (mg/g)	Lipids (mg/g)	Protein (mg/g)	Water (mg/g)
A	RACOL^®^-NF SemiSolid for Enteral Use	EN Otsuka Pharmaceutical Co., Ltd	1	5.8 - 6.3	-	6,500 - 12,500 ^a^	-	156.2	-	22.3	43.8	760
B	Hine_®_ Jerry AQUA	Otsuka Pharmaceutical Factory, Inc	0.8	6.7	-	approximately 6,000	125.6	-	9.2	18	40	808
C	Mermed Plus_®_	Terumo Corporation	0.75	6.8	283	35^a^	102	93.8	-	28.5	30	885
D	Medif_®_ Push Care_®_2.5	Nestlé Health Science	2.5	3.8	-	-	350	320	-	70	117.5	425
E	ISOCAL^®^SemiSolid Support	Nestlé Health Science	2	3.6	-	-	-	230	-	80	72	660
F	PG Soft EJ_TM_	Terumo Corporation	1.5	less than 4.0	400	20,000	241	235.5	5.5	33	60	655
G	PG Soft A_TM_	Terumo Corporation	0.75	less than 4.0	360	20,000	128.5	118	10.5	16.5	30	825
H	PG Soft A MP_TM_	Terumo Corporation	0.75	less than 4.0	460	20,000	129	117.8	11.3	18.8	24.8	825
I	F2 Shot EJ_TM_	Terumo Corporation	1	less than 4.0	470	2,000	169.5	154.5	15.0	22	40	770

^a^Measurement condition was at 20 °C

-No data

### Culture methods and sampling

A bacterial solution was added to each enteral nutritional supplement to yield a concentration of 10^2^ - 10^3^ CFU/g. The samples were allowed to incubate at 25 °C under light shielding. After 0, 4, 8, and 24 h, the number of viable cells was counted using the colony coefficient method. Nutrient agar was used as a medium for bacteria and Sabouraud medium was used for fungi.

### Enumeration of viable cells

Based on other studies of microbial growth [17–19], the data obtained in this study were not analyzed statistically because the biological significance of these types of data is considered acceptable without statistical analysis.

## Results

**Fig. 1:**
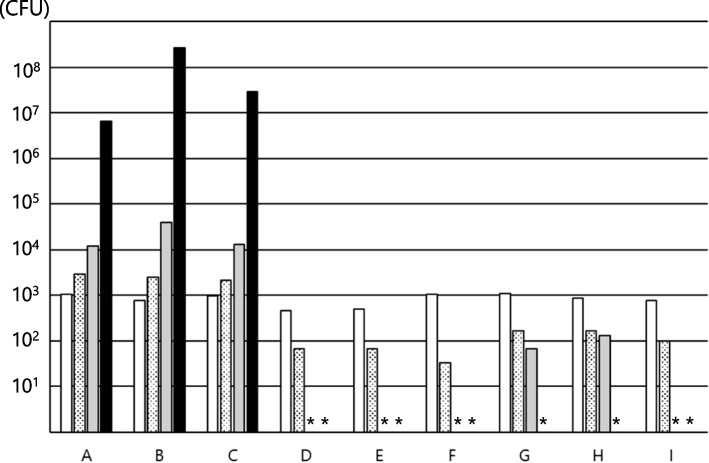
Growth of *E. coli* in various enteral nutrition products. **A** to **I** shows the growth in the following enteral nutritional supplements: **A** RACOL^®^-NF SemiSolid for Enteral Use, **B** Hine_®_ Jerry AQUA, **C** Mermed Plus_®_, **D** Medif_®_ Push Care_®_ 2.5, **E** ISOCAL^®^ SemiSolid Support, **F** PG soft EJ_TM_, **G** PG soft A_TM_, **H** PG Soft MP_TM_, and **I** F2 Shot EJ_TM_. The measurement time was as follows:  . *indicates undetectable levels

**Fig. 2:**
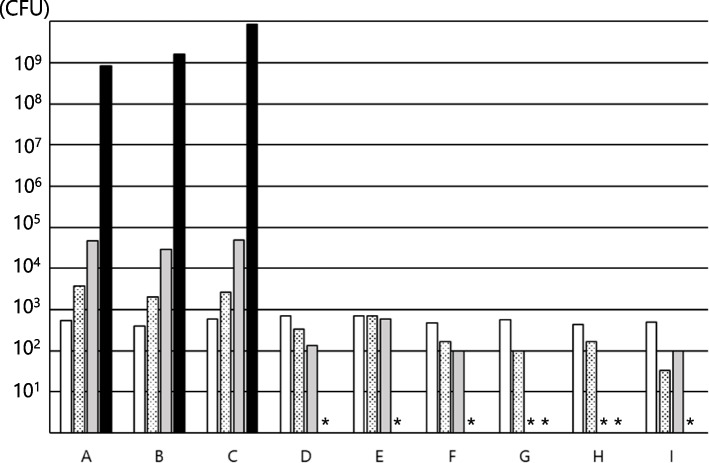
Growth of *S. marcescens* in various enteral nutrition products. **A** to **I** shows the growth in the following enteral nutritional supplements: **A** RACOL^®^-NF SemiSolid for Enteral Use, **B** Hine_®_ Jerry AQUA, **C** Mermed Plus_®_, **D** Medif_®_ Push Care_®_ 2.5, **E** ISOCAL^®^ SemiSolid Support, **F** PG soft EJ_TM_, **G** PG soft A_TM_, **H** PG Soft MP_TM_, and **I** F2 Shot EJ_TM_. The measurement time was as follows:  .* indicates undetectable levels

**Fig. 3:**
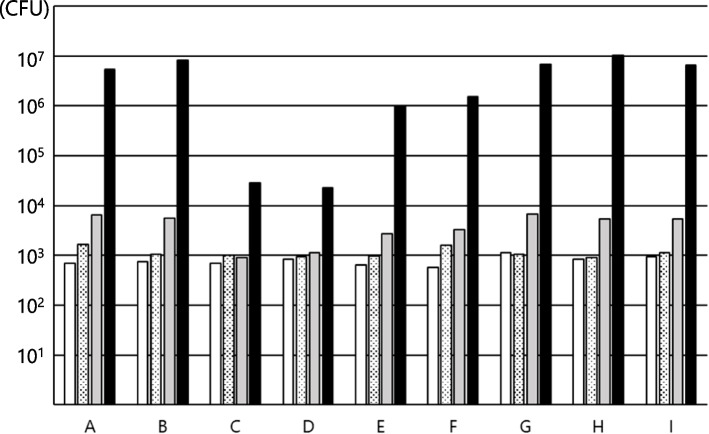
Growth of *C. albicans* in various enteral nutrition products. **A** to **I** shows the growth in the following enteral nutritional supplements: **A** RACOL^®^-NF SemiSolid for Enteral Use, **B** Hine_®_ Jerry AQUA, **C** Mermed Plus_®_, **D** Medif_®_ Push Care_®_ 2.5, **E** ISOCAL^®^ SemiSolid Support, **F** PG soft EJ_TM_, **G** PG soft A_TM_, **H** PG Soft MP_TM_, and **I** F2 Shot EJ_TM_. The measurement time is as follows:

The results are shown in Figs. 1, 2 and 3. *E. coli* grew from 1.0 × 10^3^ CFU/g at 0 h to 3.0 × 10^3^ CFU/g at 4 h, 1.2 × 10^4^ CFU/g at 8 h, and 6.5 × 10^6^ CFU/g at 24 h in RACOL^®^-NF SemiSolid for Enteral Use. It also grew in Hine_®_ Jerry AQUA from 7.7 × 10^2^ CFU/g at 0 h to 2.7 × 10^8^ CFU/g after 24 h, and in Mermed Plus_®_ from 9.7 × 10^2^ CFU/g at 0 h to 2.9 × 10^7^ CFU/g after 24 h. In contrast, for Medif_®_ Push Care_®_ 2.5, *E. coli* grew to 4.7 × 10^2^ CFU/g at 0 h, decreased to 6.7 × 10^1^ CFU/g after 4 h, and was below the detection limit after 8 h. Similarly, Isocal^®^ Semi-Solid Support, PG Soft EJ_TM_ and F2 Shot EJ_TM_ had levels below the detection limit after 8 h. In addition, *E. coli* did not proliferate, even in PG Soft A_TM_, and PG Soft A MP_TM_, and was below the detection limit after 24 h.

*S. marcescens* grew from 5.3 × 10^2^ CFU/g at 0 h to 3.7 × 10^3^ CFU/g at 4 h, 4.6 × 10^4^ CFU/g at 8 h, and 8.4 × 10^8^ CFU/g at 24 h in RACOL^®^-NF SemiSolid for Enteral Use. For Heine_®_ Jelly AQUA, the growth rate was 4.0 × 10^2^ CFU/g at 0 h, but increased to 1.6 × 10^9^ CFU/g after 24 h. For Mermed Plus_®_, the growth rate was 6.0 × 10^2^ CFU/g at 0 h, but increased to 8.4 × 10^9^ CFU/g after 24 h. For other enteral nutrients, a decrease without proliferation was observed over 24 h.

In contrast, *C. albicans* grew from 7.0 × 10^2^ CFU/g at 0 h to 1.6 × 10^3^ CFU/g at 4 h, 6.6 × 10^3^ CFU/g at 8 h, and 5.4 × 10^6^ CFU/g at 24 h in RACOL^®^-NF SemiSolid for Enteral Use. All other enteral nutrients yielded similar results with proliferation over 24 h in all enteral nutrients. PG Soft A MP_™_, which exhibited a particularly high rate of increase, grew from 8.3 × 10^2^ CFU/g at 0 h to 1.0 × 10^7^ CFU/g at 24 h.

## Discussion

Complications, such as gastroesophageal reflux disease, aspiration pneumonia, and diarrhea, are problems associated with liquid EN products in the past; however, the use of semi-solid EN products has been reported can circumvent these complications [3–8]. In addition, semi-solid EN can be administered over a short time [9], which facilitates physiological movement of the stomach and gastrointestinal tract function [4–8], and its slow absorption has been reported to help prevent hyperglycemia [20] and pressure ulcers [21]. However, semi-solidified enteral nutrients are more viscous compared with liquid nutrients, which may cause infection if retained in the catheter lumen. Various manufacturers currently market semi-solid EN products with different ingredients and physical properties. Semi-solid EN products that stay in the catheter lumen vary adheres to the catheter after injection, which depends on their physical properties [22]. In recent years, Japan has switched to small-diameter connector products for EN administration that comply with the International Standard ISO80369-3 to prevent misconnection with infusion lines; however, the complicated structure of these connectors raises concerns with respect to microbial contamination [23] and proper handling is required. Because bacterial growth due to improper handling associated with liquid EN [24–27] and subsequent infections [28, 29] have been reported, we consider that semi-solidified EN products with high viscosity are more likely to cause infection. Because no effective method has yet been established, infection control is necessary when handling enteral feeding catheters [30, 31]; however, current guidelines [3] do not provide detailed control methods and are not standardized. As a result, pharmacists must provide necessary information and guidance based on their knowledge of the proper use of drugs, but the handling of enteral nutritional supplements is not well-established [32].

Our study aimed to investigate how the characteristics of EN products affect the growth of microorganisms. The bacteria *E. coli*, *S. marcescens*, and the fungus *C. albicans*, which can be problematic in the treatment of infectious diseases, were evaluated.

The results indicated that the growth of *E. coli* and *S. marcescens* increased over time from 0 to 24 h in RACOL^®^-NF SemiSolid for Enteral Use, Hine_®_jerry AQUA, and Mermed Plus_®_. Bacterial growth depends on the nature of the bacterial species as well as composition, pH, and osmotic pressure [33], which suggests that the neutral range pH of EN may be a factor in bacterial growth. In total nutrient admixture (17.6% glucose, 5% amino acids, 4% lipid; pH 5.6, osmolality 1778) infusions, it was reported that many bacteria such as *S. aureus*, *S. marcescens*, and *Pseudomonas aeruginosa* did not grow, but only *C. albicans* and two isolates of *Staphylococcus saprophyticus* did [34]. It was also reported that bacteria such as *S. aureus*, *S. marcescens* and *Bacillus cereus* cannot grow in total parenteral nutrition (TPN) solutions without lipid due to the acidity (pH5.6 or lower), but *C. albicans* can grow regardless acidity [35]. Furthermore, it has been reported that neutral range pH, low osmolarity peripheral parenteral nutrition solutions are better environment for the growth of various microorganisms than low pH, high osmolarity TPN solutions [17]. All of the enteral nutrients in which no growth was observed had a pH value less than pH 4, indicating that pH exhibited a marked influence on the differences in bacterial growth in the enteral nutrient products. In addition, the viscosity and calorie content in which growth was observed varied, suggesting that these factors have little effect on bacterial growth. In contrast, when *C. albicans* was present, growth was observed in all nine types of EN products. Almost all fungi have been reported to be able to grow at low pH [36]. The fact that *C. albicans* can raise environmental pH at low pH [37] suggests that *C. albicans*, unlike bacteria, can grow at low pH, regardless of the composition of the enteral nutritional supplements. In this study, we did not use *S. aureus* because other studies have reported its growth in EN [15, 16]. However, further examination for *S. aureus* would be helpful to characterize microbial growth in EN.

The guidelines [3] state that administration of EN other than RTH preparations should be completed within 8 h after opening, and within 24 h for RTH preparations. However, our study revealed that bacterial and fungal growth increased slowly during the first 8 h and then rapidly over the next 24 h. RTH preparations are considered effective at preventing contamination in enteral feedings [12], but even with RTH preparations, the risk of infection may increase with inappropriate use, such as dilution of nutritional supplements or inadequate management of enteral feeding catheters. Contamination of enteral feeding catheters, which can cause blockage, may be caused by viscous enteral feedings, such as semi-solid EN, inadequate cleaning of the catheter resulting in residue on the catheter, and improper administration through the catheter. For drug administration, caution is required because there are many cases in which changes in the combination of EN supplements and pharmaceuticals may occur. Even with RTH preparations, the risk of infection can be reduced by prompt discontinuation of use after opening the package. In general, when using an enteral feeding catheter to administer enteral nutritional supplements and drugs, it should be thoroughly washed with 20 to 30 mL of water before and after use. In nursing homes and home medical care sites, which have increased in recent years, catheter management methods have become dependent upon caregivers, and it is unclear whether appropriate management is being performed.

From the results of this study, we found that if microorganisms contaminate enteral nutrients, even RTH preparations with a low infection risk, they may proliferate if the product is not used appropriately. Importantly, low-pH preparations can inhibit bacterial growth. In addition, because microbial growth is a concern regardless of physical properties, it is necessary to gather evidence so that appropriate management can be implemented regarding the handling of EN supplements and catheters.

## Conclusion

From this study, we believe that low pH can help to prevent microbial growth. However, If bacteria or fungi are introduced into semi-solid EN supplements for any reason, they will proliferate, and proper management, including adequate cleaning of the catheter lumen, is necessary to prevent infection.

## Data Availability

All data generated or analyzed during this study are included in this article. Further enquiries can be directed to the corresponding author.
